# Efficacy of Hypoxia-Inducible Factor Prolyl Hydroxylase Inhibitor on Clinical Parameters in Patients with Heart Failure

**DOI:** 10.3390/medicina60010084

**Published:** 2024-01-01

**Authors:** Mayu Yazaki, Takeru Nabeta, Yu Takigami, Yuko Eda, Teppei Fujita, Yuichiro Iida, Yuki Ikeda, Shunsuke Ishii, Junya Ako

**Affiliations:** Department of Cardiovascular Medicine, Kitasato University School of Medicine, 1-15-1 Kitasato, Minami-ku, Sagamihara 252-0329, Kanagawa, Japan

**Keywords:** anemia, hypoxia-inducible factor prolyl hydroxylase inhibitor, heart failure, chronic kidney disease

## Abstract

*Background and Objectives:* Hypoxia-inducible factor prolyl hydroxylase (HIF-PH) inhibitors have been approved as an oral drug for treating anemia in chronic kidney disease (CKD). However, the clinical effect of HIF-PH inhibitors in patients with heart failure (HF) is unclear. Thus, this study investigated the effect of HIF-PH inhibitors in patients with HF and CKD. *Materials and Methods:* Thirteen patients with HF complicated by renal anemia who were started on vadadustat were enrolled. Clinical parameters were compared before and 1 month after vadadustat was started. *Results:* The mean left ventricular ejection fraction was 49.8 ± 13.9%, and the mean estimated glomerular filtration rate was 29.4 ± 10.6 mL/min/1.73 m^2^. The hemoglobin level was significantly increased (9.7 ± 1.3 mg/dL vs. 11.3 ± 1.3 mg/dL, *p* < 0.001), and the N-terminal prohormone of B-type natriuretic peptide was significantly decreased after the introduction of vadadustat [4357 (2651−15182) pg/mL vs. 2367 (1719−9347) pg/mL, *p* = 0.002]. Furthermore, the number of patients with New York Heart Association functional class ≥ 3 was also decreased after the introduction of vadadustat [8 (61.5%) vs. 1 (7.7%), *p* = 0.008]. No thromboembolic adverse events or new tumors were observed in any patient during the study period. *Conclusions:* The introduction of vadadustat in patients with HF complicated by renal anemia led to improvements in anemia and symptoms of HF.

## 1. Introduction

Anemia is a major comorbidity in patients with chronic heart failure (CHF), occurring in 17–35% of patients with CHF [1,2,3]. The incidence of all-cause death and HF admission gradually increases as serum hemoglobin (Hb) levels fall below 13.0 g/dL in males and 12.0 g/dL in females [1]. The combination of chronic kidney disease (CKD) with HF and anemia is associated with increased mortality or progression to end-stage renal disease [4].

The relationships among anemia, HF, CKD, and death are very complicated. Anemia has several causal mechanisms. For example, primary hematologic diseases, nutritional deficiencies (primarily iron), chronic inflammation, CKD, bleeding, or fluid retention. Renal anemia causes relative erythropoietin deficiency combined with impaired iron absorption and utilization. Accordingly, the treatment of anemia in CKD includes the use of erythropoiesis-stimulating agents and iron supplementation [5]. In a previous study, darbepoetin alfa failed to reduce all-cause death and hospitalization for HF and increased the risk of thromboembolism in patients with HF and anemia [6]. Hence, few anemia therapies can improve outcomes in patients with HF and CKD.

Cardio-renal anemia syndrome describes a condition in which CHF and anemia are themselves major contributors to the deterioration of renal function and that CHF, CKD, and anemia form a harmful cycle. The three conditions form a vicious circle, in which each condition is capable of causing or being caused by another. Anemia can increase the severity of CHF and is associated with a rise in mortality, hospitalization, and malnutrition [4]. Hypoxia-inducible factor (HIF) prolyl hydroxylase (PH) inhibitors are a new class of agents for the treatment of anemia in CKD. Given the biology of the HIF pathway, it is probable that targeting the PH domain enzymes will lead to pleiotropic effects. HIF is a key transcription factor that produces a physiologic response to reduced tissue oxygen levels by activating the expression of certain genes. HIF-PH inhibitors lead to endogenous erythropoietin production and enhance iron availability to the erythron. HIF also regulates iron metabolism and handling. HIF upregulates transferrin, ceruloplasmin, and transferrin receptor 1, the latter facilitating increased plasma transport of iron to tissues. Erythropoietin production induced by HIF leads to the production by erythroblasts of erythroferrone, which limits the gene expression of liver hepcidin [7]. In a phase III trial, vadadustat was noninferior to darbepoetin alfa in increasing and maintaining Hb within the target range up to week 52 and was generally well tolerated in patients with non-dialysis-dependent CKD [8]. However, the clinical effect of HIF-PH inhibitors in patients with HF complicated by renal anemia is unclear. Thus, this study investigated the effect of HIF-PH inhibitors in patients with HF and CKD.

## 2. Materials and Methods

### 2.1. Study Subjects

This single-center retrospective observational study assessed patients with HF complicated by renal anemia who were started on vadadustat at Kitasato University Hospital between June 2021 and March 2022. All patients had stable HF with the introduction of optimal medical therapies. Patients who were followed for 1 month after the introduction of vadadustat were included in the study. Although several HIF-PH inhibitors are available in Japan, we only included the patients who introduced vadadustat, considering the differences between drugs. Patients with CKD who were not on hemodialysis and had an estimated glomerular filtration rate (eGFR) of <60 mL/min/1.73 m^2^ were eligible. There were no changes in other HF medications during the follow-up period. The patients who underwent a red blood cell transfusion in the week before and the one after data extraction were excluded. Patients who had a history of malignancy or retinopathy were also excluded. The Ethics Committee of Kitasato University Medical approved this study and the use of clinically acquired data. The need for written informed consent was waived due to the retrospective study design.

### 2.2. The Introduction of HIF-PH Inhibitors

Renal anemia was diagnosed when CKD alone was the primary cause of anemia and there were no other diseases causing anemia. A peripheral blood test, biochemistry, and vitamin/hormone measurements were performed as screening tests. A fecal occult blood test was performed to rule out gastrointestinal bleeding. Anemia was defined as Hb < 13.5 g/dL in males and <11.5 g/dL in females. We initiated treatment for renal anemia when the Hb level was <11 g/dL in several test results. If the Hb level exceeded 12 g/dL, a dose reduction was performed [9]. The starting dose of vadadustat was 300 mg orally once daily. Oral iron supplementation was initiated in patients with iron deficiency, defined as a serum ferritin level < 100 ng/mL or transferrin saturation < 20% [10].

### 2.3. Clinical Measurement and Observation

The baseline clinical evaluations included measurements of blood pressure and New York Heart Association (NYHA) functional class as well as performing laboratory tests, electrocardiography, and echocardiography upon vadadustat initiation. On echocardiography, the left ventricular ejection fraction was measured as the difference between the end-diastolic volume and the end-systolic volume divided by the end-diastolic volume. Cardiac events were obtained from the medical records. The primary endpoint was the change in clinical parameters after 1 month of vadadustat treatment. Safety endpoints were the presence of embolic and thrombotic events, such as myocardial infarction, cerebrovascular insufficiency, deep-vein thrombosis, worsening HF, or new tumors.

### 2.4. Statistical Analysis

Continuous variables were expressed as the mean ± standard deviation (SD) or as the median (interquartile range) if not normally distributed. Categorical variables were reported as counts and percentages. To compare the differences in clinical variables before and after the introduction of vadadustat, the paired-sample *t*-test was used for normally distributed continuous variables, the Wilcoxon signed-rank test was used for non-normally distributed continuous variables, and the McNemar test was performed for categorical variables. A two-tailed *p* value of <0.05 was accepted as indicative of statistical significance. JMP version 14 (SAS Institute, Cary, NC, USA) was used to perform all statistical analyses.

## 3. Results

### 3.1. Baseline Characteristics

A total of 13 patients met the inclusion criteria for this study. The baseline characteristics are shown in Table 1. The mean age was 75.4 ± 4.6 years, and there were six males (46.1%). With regard to primary heart disease, six patients (46.1%) were ischemic, and seven patients (53.9%) were non-ischemic. Eight patients (61.5%) were NYHA functional class ≥ 3. The mean systolic blood pressure was 109.5 ± 18.3 mmHg, and the mean diastolic blood pressure was 58.5 ± 9.2 mmHg. Ten patients (76.9%) were taking beta-blockers; ten patients (76.9%) were taking angiotensin-converting enzyme inhibitors/angiotensin-receptor blockers/angiotensin receptor neprilysin inhibitors; seven patients (53.8%) were taking mineralocorticoid receptor antagonists; and seven patients (53.8%) were taking sodium-glucose cotransporter 2 inhibitors. The mean eGFR was 29.4 ± 10.6 mL/min/1.73 m^2^, the mean Hb level was 9.7 ± 1.1 mg/dL, the median N-terminal prohormone of B-type natriuretic peptide (NT-proBNP) was 4357 (2651–15,182) pg/mL, and the mean ferritin level was 78.5 (16.3–270.5) ng/mL. The mean left ventricular ejection fraction was 49.8 ± 13.9%, and the mean cardiac output was 4.1 ± 1.4 L/min. There were three patients with a left ventricular ejection fraction less than 40%. All patients were newly started on vadadustat, and none were switched from an erythropoiesis-stimulating agent. Six patients (46.2%) were taking oral iron supplements.

### 3.2. Changes in Clinical Parameters

**Figure 1:**
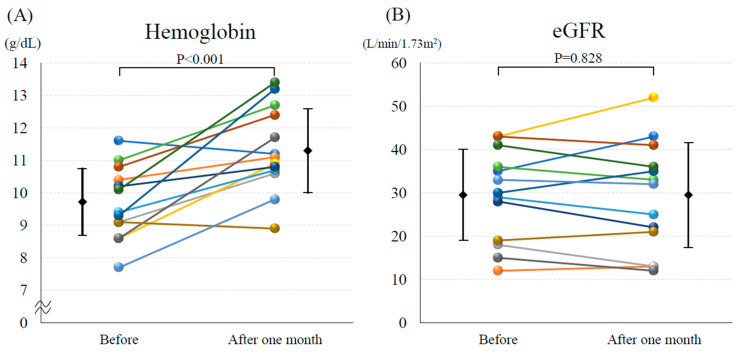
Changes in (**A**) hemoglobin level and (**B**) estimated glomerular filtration rate (eGFR) before and 1 month after the introduction of vadadustat.

**Figure 2:**
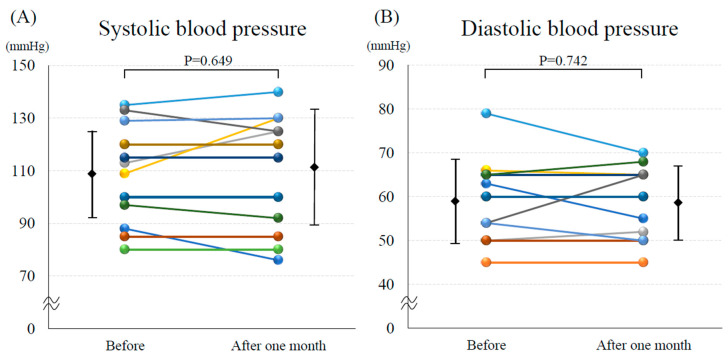
Changes in (**A**) systolic blood pressure and (**B**) diastolic blood pressure before and 1 month after the introduction of vadadustat.

**Figure 3:**
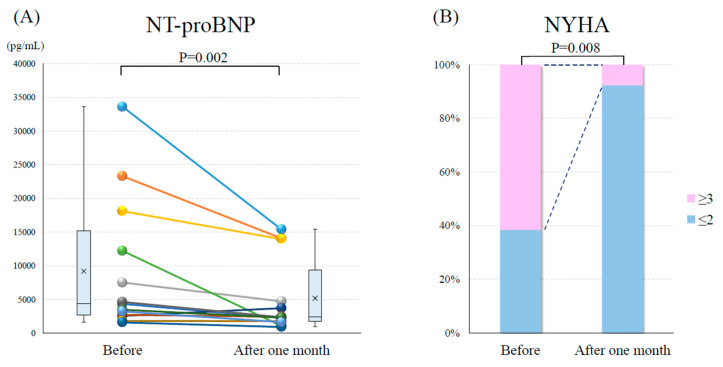
Changes in the (**A**) N-terminal prohormone of B-type natriuretic peptide (NT-proBNP) and (**B**) New York Heart Association (NYHA) functional class before and 1 month after the introduction of vadadustat.

The mean Hb level significantly increased after the introduction of vadadustat (9.7 ± 1.1 vs. 11.3 ± 1.3 mg/dL, *p* < 0.001; Figure 1A). The mean eGFR did not change after the introduction of vadadustat (29.4 ± 10.6 vs. 29.1 ± 12.6 mL/min/1.73 m^2^, *p* = 0.828; Figure 1B). Both systolic blood pressure (109.5 ± 18.3 vs. 110.6 ± 21.4 mmHg, *p* = 0.649; Figure 2A) and diastolic blood pressure (58.5 ± 9.2 vs. 58.1 ± 8.2 mmHg, *p* = 0.742; Figure 2B) did not change before and after the introduction of vadadustat. The median NT-proBNP was significantly decreased after the introduction of vadadustat [4358 (2651–15,182) vs. 2367 (1719–9347) pg/mL, *p* = 0.002; Figure 3A]. Moreover, the number of patients with NYHA functional class ≥ 3 significantly decreased after the introduction of vadadustat [8] (61.5%) vs. 1 (7.7%), *p* = 0.008; Figure 3B]. In seven patients, echocardiography was performed 1 month after the introduction of vadadustat. In these patients, there were no significant changes in left ventricular ejection fraction after the introduction of vadadustat (49.8 ± 13.9 vs. 43.8 ± 19.1%, *p* = 0.965).

### 3.3. Adverse Events

There were no adverse events (worsening HF, embolic and thrombotic events, or new tumors) in the study patients during this period.

## 4. Discussion

### 4.1. Major Findings

This study highlighted changes in clinical parameters after the introduction of vadadustat in patients with HF complicated by renal anemia. A comparison of data before and 1 month after the introduction of vadadustat revealed a significant increase in Hb levels and a decrease in NT-proBNP levels. The NYHA functional class was also improved after the introduction of vadadustat. No adverse events were reported during the observation period.

### 4.2. Improvement of Anemia with HIF-PH Inhibitors

The pathogenesis of anemia in HF is multifactorial. Functional iron deficiency due to chronic inflammation, decreased erythropoietin production due to renal dysfunction, gastrointestinal bleeding, and fluid dilution all contribute to anemia through complex interactions [11]. In a previous study, erythropoiesis-stimulating agents could not improve cardiac events in patients with renal anemia and HF [6]. The reason can be partially explained by the poor response to erythropoiesis-stimulating agents in patients with HF and CKD. The poor response to erythropoiesis-stimulating agents was shown by a lower increase in Hb after an erythropoiesis-stimulating agent was introduced. In addition, the rates of cardiovascular events have previously been shown to be higher in patients with a poor response to erythropoiesis-stimulating agents than in patients with a better response [12]. A previous meta-analysis demonstrated that the impaired erythropoietin response was highly prevalent in patients with HF [13].

The present study results showed that HIF-PH inhibitors can potentially improve renal anemia in most patients with HF and CKD. Erythropoietin production induced by HIF leads to the production by erythroblasts of erythroferrone, which limits the gene expression of liver hepcidin. HIF also regulates iron metabolism and handling [7]. HIF-PH inhibitors increase Hb levels through a pathway different from that of conventional erythropoiesis-stimulating agents. Therefore, we consider that HIF-PH inhibitors are a potentially new class of anemia drugs for treating patients with HF complicated by renal anemia who are also intolerant to conventional therapy, including erythropoiesis-stimulating agents.

### 4.3. Improvement of Heart Failure Symptoms with HIF-PH Inhibitors

In this study, an improvement in NYHA functional class was observed after treatment with HIF-PH inhibitors. We expect that the improvement in HF symptoms was due to the increase in Hb levels. In a previous study, quality of life and vital status such as physical functioning, social functioning, bodily pain, and general health improved only in the higher Hb level group after an erythropoiesis-stimulating agent was introduced for treating patients with CKD [14]. In patients with HF, correction of anemia was previously shown to reduce afterload and improve HF symptoms [15].

This study also showed that NT-proBNP levels decreased after the introduction of the HIF-PH inhibitor. This result agreed with that of a previous case report, which showed that the NT-proBNP level significantly decreased in patients with HF and CKD after the introduction of an HIF-PH inhibitor [16]. Some animal studies have demonstrated that HIF-PH inhibitors could improve cardiac function or show a cardio-protective effect in patients with ischemic heart disease [17,18]. Therefore, this cardio-protective effect derived from HIF-PH inhibitors might also be related to a reduction in NT-proBNP levels in patients with HF. In contrast with the present study, Nakamura et al. [19] showed that NT-proBNP levels were not significantly changed in patients with HF and CKD after 3 months from the introduction of HIF-PH inhibitors, despite an increase in Hb levels. This discrepancy might be explained by differences in the study cohort and an improvement in Hb level [from 9.7 mg/dL to 11.3 mg/dL vs. 9.6 mg/dL to 10.7 mg/dL]. Given that there are still little data on the effect of HIF-PH inhibitors in patients with HF, this is a subject for future research.

### 4.4. Adverse Events

It has previously been noted that erythropoiesis-stimulating agents can increase blood pressure [20]. Increasing peripheral vascular resistance by reducing blood viscosity and reducing hypoxic vasodilation is the main mechanism for increasing blood pressure with erythropoiesis-stimulating agent treatment [21,22]. Although there could be similar concerns about increasing blood pressure with HIF-PH inhibitors [10], we did not observe a significant increase in blood pressure after the introduction of HIF-PH inhibitors in the present study. Therefore, caution regarding blood pressure may not be necessary with HIF-PH inhibitor treatment.

There were no adverse events, such as worsening HF, thromboembolism, or new tumor occurrences, during the study period. A previous randomized study showed that rates of adverse events were similar between patients with CKD and those treated with vadadustat or darbepoetin alfa [23]. However, HIF and vascular endothelial growth factor are involved in retinal vascular development [24]. Therefore, the effects of HIF downstream genes on angiogenesis, especially vascular endothelial growth factor production, require careful attention. Long-term observation would be necessary to determine any new onset and progression of malignancy and retinopathy.

### 4.5. Clinical Implications

The introduction of HIF-PH inhibitors improved anemia in patients with HF complicated by renal anemia. These inhibitors might be expected to improve renal anemia in patients with HF who have underlying chronic inflammation and do not respond to other therapies, which is especially beneficial for patients with non-dialysis-dependent CKD because HIF-PH inhibitors are oral drugs that, unlike erythropoiesis-stimulating agents, do not require injections.

### 4.6. Study Limitations

Several limitations need to be acknowledged in this study. First, this study was a single-center, retrospective, observational, small patient population study, which possibly resulted in a patient selection bias and lower statistical power. Second, only some of the patients had echocardiographic measurements after 1 month, so we could not observe the change in all patients. Third, we did not obtain any other sensory measurements of exercise capacity. Fourth, although there were no changes in other HF medications during the follow-up period, it cannot be ruled out that other drugs may have affected the improvement of HF symptoms.

## 5. Conclusions

After the introduction of HIF-PH inhibitors in patients with HF complicated by renal anemia, significant improvement in anemia and symptoms of HF was observed. These results indicate that HIF-PH inhibitors could potentially be an effective treatment option for patients with HF complicated by renal anemia.

## Figures and Tables

**Table 1:** Baseline characteristics.

Variables	N = 13
Age (years)	75.4 ± 4.6
Male, n (%)	6 (46.1)
Body surface area (m^2^)	1.49 ± 0.16
Primary disease, n (%)	
Ischemic heart disease	6 (46.1)
Valvular heart disease	5 (38.5)
Dilated cardiomyopathy	1 (7.7)
Adult congenital heart disease	1 (7.7)
NYHA functional class ≥3, n (%)	8 (61.5)
Systolic blood pressure (mmHg)	109.5 ± 18.3
Diastolic blood pressure (mmHg)	58.5 ± 9.2
Heart rate (beats/min)	73.7 ± 13.3
Comorbidity, n (%)	
Diabetes mellitus	3 (23.0)
Hypertension	7 (53.8)
Hyperlipidemia	6 (46.2)
Medications, n (%)	
Beta-blocker	10 (76.9)
ACEi/ARB/ARNI	10 (76.9)
Mineralocorticoid receptor antagonist	7 (53.8)
Sodium-glucose cotransporter 2 inhibitor	7 (53.8)
Furosemide	8 (61.5)
Statin	9 (69.2)
Electrocardiogram, n (%)	
Sinus	6 (46.2)
Atrial fibrillation	2 (15.4)
Pacemaker	5 (38.5)
Laboratory values	
Hemoglobin (mg/dL)	9.7 ± 1.1
Serum creatinine (mg/dL)	1.79 ± 0.82
eGFR (mL/min/1.73 m^2^)	29.4 ± 10.6
NT-proBNP (pg/mL)	4357 (2651−15182)
Ferritin (ng/mL)	78.5 (16.3−270.5)
Transferrin saturation (%)	33.1 ± 21.6
Echocardiography	
Left ventricular end-diastolic dimension (mm)	52.9 ± 7.0
Left ventricular end-systolic dimension (mm)	37.9 ± 9.9
Left ventricular ejection fraction (%)	49.8 ± 13.9
Cardiac output (L/min)	4.1 ± 1.4
E/e′	16.7 ± 8.5
Transtricuspid pressure gradient (mmHg)	32.9 ± 12.6
Mitral regurgitation grade ≥3, n (%)	6 (46.2)

Abbreviations: ACEi/ARB/ARNI, angiotensin-converting enzyme inhibitor/angiotensin-receptor blocker/angiotensin receptor neprilysin inhibitor; eGFR, estimated glomerular filtration rate; NT-proBNP, N-terminal prohormone of B-type natriuretic peptide; NYHA, New York Heart Association.

## Data Availability

The data presented in this study are available on request from the corresponding author.
